# Insights in bone marrow failure syndromes: take home messages from the 3rd ESH-EBMT-EHA-IPIG translational research conference

**DOI:** 10.1038/s41409-025-02710-2

**Published:** 2025-09-20

**Authors:** Fabiana Cacace, Marthinus J. Dicks, Pablo Siliceo

**Affiliations:** 1 AORN Santobono Pausilipon, Department of Hematology and Oncology, Hematopoietic Cell Transplantation and Cellular Therapy Unit, Naples, Italy; 2 Duke University, Pediatric Transplantation and Cellular Therapy, Durham NC, USA; 3 Severe Aplastic Anaemia Working Party of European Society for Blood and Marrow Transplantation, Leiden, The Netherlands; 4 Division of Haematology, Groote Schuur Hospital, Cape Town, South Africa; 5 Division of Haematology, University of Cape Town, Cape Town, South Africa; 6 Departamento de Medicina Genómica y Toxicología Ambiental, Universidad Nacional Autónoma de México, Mexico City, Mexico; 7 Laboratorio de Falla Medular y Carcinogénesis, Instituto Nacional de Pediatría, Mexico City, Mexico

**Keywords:** Stem-cell research, Cell signalling, Myelodysplastic syndrome, Acute myeloid leukaemia

## Abstract

Bone marrow failure (BMF) syndromes (BMFSs) are a group of disorders characterized by the bone marrow’s inability to produce sufficient blood cells. Over the years, diagnostic and therapeutic approaches have significantly evolved, now progressing towards personalized treatment strategies. Traditionally, these disorders include both acquired forms and inherited conditions. The inherited conditions, in particular, are becoming increasingly complex, with new genetically-defined BMFSs being identified beyond the well-known Fanconi Anemia (FA), Dyskeratosis Congenita (DKC), Diamond–Blackfan Anemia (DBA) and Shwachman–Diamond syndrome (SDS). A dedicated conference, supported by leading international scientific networks, such as the European School of Hematology (ESH), European Bone Marrow Transplantation (EBMT), European Hematology Association (EHA), and the International PNH Interest Group (IPIG), was recently organized to discuss and to emphasize how our knowledge in BMFs field has improved, eventually becoming a topic of interest for general hematologists and those involved in hematopoietic cell transplantation (HCT). This manuscript highlights some key findings from this 3rd ESH-EBMT-EHA-IPIG Translational Research Conference, focusing on the pathogenic mechanisms of BMFSs and their secondary malignancies, as well as advances in pathophysiology-driven treatments for both acquired and inherited BMFSs.

## Introduction

Hematopoiesis is a hierarchical process where hematopoietic stem cell (HSC) self-renews and differentiates into mature, and functional blood cells [1]. Disruption of this process results in a group of heterogenous diseases, commonly known as bone marrow failure (BMF) syndromes (BMFSs). Here, either acquired or inherited factors can impair hematopoietic function, leading to insufficient blood cell production.

In inherited BMFSs, abnormal differentiation and maturation of one or more hematopoietic lineages are caused by germline genetic abnormalities that impair several critical cellular functions. These include DNA repair (e.g., Fanconi anemia (FA)), telomere maintenance (e.g., Dyskeratosis Congenita (DKC)), HSC differentiation and self-renewal (e.g., GATA2 deficiency), ribosomal-associated functions (e.g., Shwachman–Diamond syndrome (SDS)), or antiapoptotic signal regulation (e.g., congenital amegakaryocytic thrombocytopenia) [2].

Moreover, some inherited BMFSs may selectively affect a single hematopoietic lineage. Typical examples include congenital neutropenia (CN) and Diamond–Blackfan anemia (DBA), which affect granulopoiesis (via *ELA2* gene mutations) and erythropoiesis (via *RPS19/RPL5/TSR2* gene mutations), respectively [2].

In contrast, acquired phenotypes are caused by immune-mediated damage to the HSC pool due to external factors, such as chemical agents, drugs, or various viral infections [1–4]. In these cases, HSC damage can manifest as direct cell-mediated killing by cytotoxic T lymphocytes (CTLs) or cytokine-transduced inhibition, as demonstrated by excess production of cytokines, particularly INF-γ and TNF-α [3].

While acquired forms of BMFSs have been extensively defined, the list of inherited forms continues to grow, often involving complex syndromes beyond a single hematologic disorder [5].

## BMFSs are more than one disease: clinical challenges in the daily practice

Despite the different etiologies, discriminating between acquired and inherited BMFSs is not always straightforward. In many cases, BMF of idiopathic diseases could be the first sign of a more complex diagnosis, like in some congenital immune disorders.

Primary immunoregulatory disorders, unlike common primary immunodeficiencies (PIDs), are a group of conditions characterized by immune dysregulation and autoimmunity, rather than the increased susceptibility to infections, leading to immune-mediated attack on bone marrow precursors [6]. Examples include the autoimmune lymphoproliferative syndrome (ALPS), caused by a defective lymphocyte apoptosis mediated by the Fas/Fas ligand pathway [7], and deficiencies in ADA2 (DADA2), an enzyme involved in purine metabolism, both cases having impairment of marrow precursors and immune system function [8, 9].

Ageing and chronic low-grade inflammation have also been identified as factors that not only drive HSC dysfunction (contributing to impaired adaptive immunity) but also increase the risk of hematological malignancies in both elderly individuals and patients with BMF (regardless of age) [10]. Similarly, small Rho-GTPase Cdc42 has been identified by Florian et al. as a key factor in cell-intrinsic ageing, actin and tubulin organization, cell adhesion and cell polarity. In fact, the activity of Cdc42 is significantly increased in both aged-HSCs and in young HSCs with ageing-like phenotypes, suggesting a critical role of this protein in modulating cellular ageing and making it an attractive pharmacological target for HSC rejuvenation [11, 12].

In other conditions, such as patients with mutations in *GATA2* or *SAMD9/SAMD9L* genes, impaired growth of hematopoietic progenitors may be initially linked to the presence of myeloid malignancies [13, 14]. These gene defects are often associated with specific, extra-hematological syndromes. Moreover, some genes, usually carrying somatic mutations in myeloid cancers, may be behind the development of inherited BMFSs when present as germline mutations. This is further complicated by the fact that cytopenia may not be the initial presentation in some patients with syndromic features [14].

These divergent characteristics make for a challenging differential diagnosis, ultimately requiring advanced techniques that go beyond clinical evaluation and dive deeper into standard laboratory testing [15]. Immunological screening, for example, has detected immune deficiencies and dysregulation in some pediatric patients with suspected aplastic anemia (AA), making it a crucial for the diagnostic workup [15]. Such advances enable more effective risk stratification and the creation of personalized treatment strategies for this heterogeneous group of disorders.

## The era of next-generation sequencing and other high-throughput genetic testing: advances in differential diagnosis, clinical monitoring, and therapeutic approach in BMFSs

The routine availability of next-generation sequencing (NGS) has significantly improved diagnostic accuracy and differential diagnosis. This has led to the identification of more genetic defects each year, revealing previously undiagnosed inherited BMFSs [16]. These technologies enable the identification of diverse gene mutations (or variants), which result in mutated proteins with a possible pathogenic effect on the biological pathways. Of note, not all variants necessarily cause a disease (or can account for a clinically-defined phenotype).

The concept of pathogenic (P) variant (V) was introduced, together with highly specialized definitions and concepts, which are summarized in a glossary (see box below) to guide clinician’s understanding [17, 18].

**Table:** 

Pathogenic variant (PV): a genetic change identified as the definitive cause of the patient’s disease.
Likely pathogenic variant (LPV): a genetic change that is highly likely to be the cause of the patient’s disease, but with some remaining uncertainty. This information should be used with caution in clinical decision-making due to the potential for ambiguity.
Variant of uncertain significance (VUS): a genetic change that may contribute to the disease, but there is insufficient or conflicting evidence to confirm its role. These Vs are typically very rare, predicted to be harmful, and the gene is associated with the patient’s condition, though further evidence is needed.
Null variant (NV): a genetic change that leads to the absence of a gene product. The gene product is either undetectable at the molecular level or fails to function at the phenotypic level.
Clonal hematopoiesis of indeterminate potential (CHIP): is characterized by the presence of at least one clinically relevant somatic mutation commonly associated with myelodysplastic syndrome (MDS) or other myeloid neoplasms, without persistent cytopenia. It is diagnosed after excluding MDS, other hematopoietic neoplasms, and any other underlying diseases as the cause.
Clonal cytopenia of undetermined significance (CCUS): is identified by the presence of one or more somatic mutations typically seen in myeloid neoplasms, with an allele burden of ≥2%, detected in bone marrow or peripheral blood cells. It is characterized by persistent cytopenia (≥4 months) in one or more blood cell lineages, without meeting the diagnostic criteria for a myeloid neoplasm, and after excluding all other potential causes of cytopenia and molecular abnormalities.
Idiopathic cytopenia of undetermined significance (ICUS): is characterized by persistent cytopenia in one or more blood lineages for at least 6 months, with no explanation from any other disease. The diagnostic criteria for a myeloid neoplasm are not met.

Large-scale exome/genome sequencing is shifting genetic discovery from “a phenotype-first to a genome-first” approach, refining phenotypes based on genetic markers [19]. This approach is used in biomedical databases, such as the UK Biobank and Geisinger MyCode project to identify genetic associations with diseases like liver and eye disorders, cancer, cardiovascular disease, type 2 diabetes, and obesity, aiming to uncover genotype–phenotype correlations [20, 21]. Therefore, applying this approach even to BMFSs or myeloid malignancies will allow a better assessment of global prevalence and penetrance of PV, improving risk assessment and survival [22]. A study on 404 patients with MDS and BFM, for example, found a high frequency of germline PV, in MDS group, supporting the need for comprehensive germline genetic testing for all MDS patients, regardless of age at diagnosis [23, 24].

The use of NGS is now well-established to confirm the diagnostic suspect of a variety of typical BMFSs, such as FA, DKC, DBA, and SDS [5], but it also led to the identification of novel diseases by specific gene lesions. Some examples with clinical implications, discussed during the meeting, are listed.

- *GATA2 insufficiency: the correlation genotype–phenotype may allow risk stratification of patients*.
  NV of this gene may confer earlier manifestation of disease, such as lymphoedema and sensorineural deafness, while intron 4 variants are more likely to be asymptomatic [25]. Additionally, in murine models of GATA2 insufficiency, alterations in DNA methylation and histone modifications have been described as a putative route for the development of high-risk MDS [26].
- *DKC and other telomere biology disorders (TBDs): altered molecular pathway may change therapeutic approach.*
  Şerifoğlu et al. showed, in zebrafish models, that telomere dysfunction or shortening can activate the cGAS-STING innate immune pathway, involved in DNA damage response. It increases cellular senescence and reduces proliferative capacity in the adult hematopoietic organ. The cGAS-STING pathway may play an important role in hematopoietic cell aplasia in TBDs. In particular, the knockout of the telomerase enzyme TERT (involved in telomerase maintenance) in zebrafish may lead to aplasia, whereas concurrent knockout of TERT and STING may restore hematopoietic homeostasis. These findings suggest that STING inhibitors or immune-modulating therapies could mitigate inflammation driven by telomere dysfunction in TBDs [27].
- *Overlap BMFSs and PIDs: improvement in differential diagnosis*.
  As reported above, some PIDs can present with pancytopenia [6, 8, 9, 13]. Recently, novel immunological and genetic profiles linking BMFSs to PIDs have been identified [15].
  For example, DADA2, a systemic syndrome characterized by pro-inflammatory M1 macrophage polarization, impaired generation of the memory B cell pool, and altered IgA class switching [28], can mimic conditions such as ALPS or DBA, often leading to diagnostic delays.
  Furthermore, mutations in the non-homologous end-joining (NHEJ) pathway, which plays a crucial role in repairing DNA double-strand breaks and mediating V(D)J recombination, can cause certain PIDs [29, 30]. VUSs in this pathway are associated with diseases like Nijmegen breakage syndrome and ligase IV deficiency, both of which frequently mimic AA [31].
  While low plasma ADA2 levels and elevated TNFα are diagnostic markers for DADA2, immunological screening is essential to avoid delays, especially when pancytopenia is the sole clinical presentation in disorders that impact both marrow cell development and immune function.
  Accurate and timely diagnosis is critical to guide appropriate treatment [15], particularly because patients harboring mutations in the NHEJ pathway exhibit heightened sensitivity to genotoxic therapies [29].

Outside inherited BMFSs characterized by germline variants, the use of NGS has also revolutionized the field of acquired forms. Somatic mutations are extremely frequent in all BMFSs and often lead to clonal hematopoiesis.

## Conventional and unconventional damage of HSC in BMF: insights for clinicians

Over time, the theory of AA’s etiopathogenesis has evolved, while maintaining the fundamental concept that the presence of a (neo)antigen can impair immune tolerance [3].

The environmental setting in which it occurs is complex and has led to the development of two theories regarding the etiology of AA.

The “classic” view suggests that AA is triggered by a rare viral antigen in individuals predisposed to immune hyperreactivity. The hematopoietic damage would be produced by immune activation and cytokine secretion (e.g., IFN-γ, TNF-α), as previously explained [3, 4, 32].

In contrast, the “alternative” theory proposes that an antigenic trigger induces a pathogenic response in the context of a dysregulated immune system, and auto-reactivity compensates for the lack of normal immune humoral and cytotoxic responses. This theory is supported by evidence that germline mutations associated with PIDs have been identified in immune-mediated cytopenia associated with BMF (as mentioned earlier) [15]. The hypothesis proposes that rare germline mutations which predispose individuals to PIDs may play a role in the pathogenesis of AA and also likely paroxysmal nocturnal hemoglobinuria (PNH).

In AA-HSCs, the genes involved in cytokine signal transduction, stress response, and defense/immune response are upregulated. It has been demonstrated that the presence of stressed, immunologically activated, or dying target cells, rather than an intrinsically abnormal population, contributes to the pathogenesis of AA [3, 4]. Concomitantly, clinical and experimental evidences support the idea of a T-cell-mediated immune attack on AA-HSC, confirmed by the presence of CTLs in BMF patients [4]. The clonal T-cell expansion, as identified by sequencing of the T-cell receptor repertoire, defines a spectrum of disorders known as large granular lymphocyte (LGL) disorders, usually associated with AA or PNH [16, 17]. LGL syndromes can range from reactive LGL expansion to aggressive LGL leukemia, with different clinical phenotypes (from transient immune reactions to true malignancies).

Recent data also shows the presence of somatic mutations in *STAT3* in these expanded CTLs, resulting in autoreactivity and autoimmunity (a phenomenon also described in AA) [33].

The role of PNH in BMFSs remains incompletely understood. PNH is primarily caused by a somatic mutation in the *PIGA*, a gene involved in the synthesis of glycosylphosphatidylinositol (GPI) anchors. However, BMF in PNH appears to be associated with pathogenic mechanisms similar to those involved in AA [3, 4]. According to the “escape theory”, PNH-HSCs have no chance of expanding in the presence of a vast majority of normal cells. This is confirmed by the observation that circulating PNH-cells can be detected even in healthy individuals, in the absence of clinical manifestations [34]. External conditions may, however, create a permissive environment for the expansion of the PNH clone. In the setting of an immune response guided by antigens against GPI-linked molecules on HSCs, for example, PNH-HSCs can escape this injury [4]. An oligoclonal T-cell pool, detected in PNH patients, supports this notion, as similarly seen in AA [33, 35, 36].

In summary, a persistent autoimmune attack may lead to the escape of “resistant” clones, which may manifest in various forms, ranging from PNH to mutations linked to clonal hematopoiesis.

Despite different etiologies, BMFSs share common features, such as immune dysregulation and an increased risk of clonal evolution, predisposing individuals to MDS and acute myeloid leukemia [37, 38].

## BMF and myeloid malignancies: reshaping Damashek’s riddle

“What do AA, PNH and acute leukemia have in common?” [31] This question was raised over 50 years ago, implying the intriguing theory that clonal hematopoiesis could be the minimum common denominator among these, apparently dissimilar, diseases. Indeed, based on the observation that PNH often develops with AA (or vice versa), William Dameshek laid the foundation for the definitions of BMF mechanisms and of clonal hematopoiesis.

According to the hierarchical model of hematopoiesis, primitive cells undergo mitosis to develop into mature stem cells that can terminally differentiate through stochastic or deterministic processes [39].

In the event of a diseased HSC compartment, resulting either from aborted maturation of primitive stem cells or from the loss of HSCs by both extrinsic and intrinsic insults, clonality may arise as a “consequence” of a reduced HSC pool (e.g., AA).

After an immune attack on the bone marrow, on the other hand, cells may lose their canonical ligand to CTLs and can be selectively expanded, leading to pathological conditions like PNH. Here, clonality may be considered as an “escape” mechanism.

Finally, under the influence of CTLs, some targeted cells may undergo genetic damage, leading to altered proliferation associated with chromosomal deletions and/or MDS, as a clinical manifestation. Here, clonality may act as the etiological agent [3, 39]. These clues led to the definition of AA and PNH as different responses to a bone marrow insult [39].

Alternatively, it is currently accepted that an autoimmune/inflammatory milieu, damaging the HSC compartment, is eventually associated with an increased risk of expanding individual clones carrying somatic mutations in recurrent genes [40]. This event, known as “somatic genetic rescue”, can somehow rescue hematopoiesis through the expansion of an individual clone [41], following two different clinical courses: “adaptation”, expressed as CHIP/CCUS/ICUS, or “maladaptation”, as malignant clonal evolution [42].

Therefore, the risk of clonal evolution in BMFSs towards malignant or premalignant diseases requires careful monitoring. Early detection of these conditions is now achievable through the identification of predisposing mutations and pre-malignant states [43].

Longitudinal genomic studies in inherited BMFSs have revealed that acquired somatic alterations can overlap, improving HSC function and promoting the expansion of mutated clones [44]. This type of response has been further defined in the context of SDS, a ribosomopathy caused by mutations in the SBDS and EFL1 proteins, which ultimately impair the release of the ribosome anti-association factor, eIF6, disrupting ribosome assembly. In contrast, somatic genetic alterations in SDS-HSCs, which reduce expression of eIF6 or its association with the ribosome, can suppress defects in ribosome assembly and provide a selective advantage over non-modified cells [45].

In the context of FA, instead, copy number alterations, such as 1q+, 3q+, del7/7q−, and 5q− can drive maladaptive somatic rescue contributing to malignant transformation, as well as abnormalities in genes like *RUNX1* and *RAS* [46].

While the clinical implications of this somatic compensation in inherited BMFSs are still being explored, understanding these mutational processes provides valuable insights into disease pathophysiology and future treatments. These include genetic selection mechanisms driving myeloid neoplasms in SDS through the acquisition of maladaptive TP53 mutations [47], or eIF6 suppressor mimic molecules as a promising therapeutic strategy for SDS [46], respectively.

Kulasekararaj et al. discussed the need to address risk assessment of clonal evolution in AA patients, considering different approaches at the time of diagnosis, follow-up and transformation. Mutations in AA are present at diagnosis. Similar findings were observed 6 months post-diagnosis, with an increase in mutational patterns regardless of hematological response to immunosuppressive treatment (IST). Mutational risk has also been associated with age, disease severity, and elevated neutrophil counts. However, eltrombopag treatment, as part of IST, showed no significant correlation with adverse mutational impacts (as well as mutational burden, in general) [48].

The contextualization of somatic mutations with respect to age of the patient, response to IST, time of secondary malignancy diagnosis/evolution is crucial for a better risk assessment of BMFSs (and their eventually evolution to secondary hematological malignancies). Secondary myeloid neoplasms represent the most severe long-term complications in AA and PNH. Del7/7q, and *ASXL1*, *SETBP1*, *RUNX1*, and *RAS* pathway gene variants are the most frequent mutations in AA-MDS. Nevertheless, the risk of secondary myeloid neoplasms in AA seems to be linked primarily to disease severity, treatment non-responsiveness and the patient’s age. Secondary malignancies in these patients have been described as high-risk disorders in terms of morphological, karyotypic, and molecular characteristics. This should be considered with caution in medical management [43].

## Inherited BMFs: advances in treatment approach

HCT may serve as a curative treatment for certain congenital disorders, effectively correcting hematopoietic deficiencies. However, it does not address congenital malformations or reduce the risk of developing solid tumors. Transplant strategies differ depending on the type of disorder, with careful evaluation of both hematological and extra-hematological manifestations required prior to transplantation [49].

Key points addressing transplantation as management of inherited BMFSs include the type of donor and diverse conditioning regimens’ availability. Specifically, the optimal donor is a matched sibling donor (MSD); however, it is important to screen the donor for genetic defects, as inherited BMFSs can be present with varying clinical and hematological manifestations, even within the same family. In the absence of an appropriate MSD, a matched unrelated donor (MUD) should commonly follow. Other options, such as haploidentical donors, unrelated donors, or cord blood, may also be considered.

The choice between myeloablative conditioning and reduced-intensity conditioning (RIC) regimens depends on the specific BMFS, with irradiation being avoided due to the increased risk of developing cancer. Patients with FA and DKC are advised to undergo RIC.

Long-term follow-up is mandatory due to high risk of developing secondary malignancies, extra-hematological manifestations, and iron overload.

Off-label use of drugs and other non-transplant strategies for inherited BMFSs are additional therapeutic approaches to consider when managing these disorders.

Androgens, for example, have long been used in FA and TBDs, inducing changes in gene transcription, activating EPO receptors and stimulating the activity of telomerase enzymes [50]. A recent phase 2 trial showed telomere elongation in TBD treated with nandrolone decanoate [51]. Additionally, corticosteroids have been used in DBA, with an initial response seen in 80% of patients [52]. However, navigating steroid side effects can be challenging. While the mechanism of action is not yet completely understood, steroids have been shown to elicit stressed erythropoiesis and inhibit apoptotic pathways mediated by p53, cMyc, and mTor [53]. Alternatively, growth factors, such as G-CSF, have been known to have a role in CN and are mainly used to limit infections [54]. For other disorders, such as WHIM (warts, hypogammaglobulinemia, infections, and myelokathexis) syndrome, plerixafor is a viable option and was shown to be non-inferior to G-CSF [55].

While the advancements in treatment options for inherited BMFSs are promising, they also serve to underscore the importance of an accurate diagnosis in these rare diseases.

## Treatment of AA: should the transplant be the only therapeutic approach?

The choice of frontline therapy in AA depends on disease severity, patient age, donor availability, and access to optimal treatments. Initially, a watch-and-wait approach is often adopted for milder pancytopenia. Here, we outline key points regarding different therapeutic approaches for different clinical scenarios in AA.

Guidelines for the treatment of AA have been provided by the British Society of Hematology and the American Society for Transplantation and Cellular Therapy, offering valuable insights into the evolving management of allogeneic HCT and IST.

According to the British guidelines, the standard first-line treatment for young patients (<40 years old) with newly diagnosed severe AA (SAA) is allogeneic HCT from an MSD. However, horse anti-thymocyte globulin and ciclosporin-based IST is currently considered as an alternative treatment option for SAA or very SAA (VSAA) in the absence of MSD and in older SAA/VSAA patients. MUD-HCT in adults would be recommended after IST failure and should be considered upfront for young adults with severe infections [56].

The American guidelines similarly recognized allogeneic HCT as a potentially curative treatment for SAA and strongly support fludarabine-containing regimens for patients at high risk of graft failure, particularly those receiving MUD or haploidentical donor transplants.

These guidelines further support increased utilization of HCT as upfront treatment, prioritizing MUD or haploidentical donor transplants over IST in children and young adults who lacked an MSD [57].

The Baltimore protocol [58], which utilizes a four-drug regimen for GVHD prophylaxis and incorporated total body irradiation (TBI) for conditioning, demonstrates improvement in engraftment rates while minimizing GVHD. This strategy can be proposed to improve outcomes in unrelated or elderly sibling transplants.

Making treatment decisions for these patients, especially regarding initial therapy, remains challenging, as there are no universally applicable guidelines due to disparities in access to care across different regions.

Furthermore, it is imperative to consider the pediatric perspective on the management of SAA in patients without an MSD, building upon the previously discussed guidelines [56, 57]. Some retrospective studies [59] on the use of haploidentical donor transplants in SAA patients demonstrated the superiority of this approach over IST in terms of OS and event-free survival (EFS) [60]. Additionally, outcomes with haploidentical donor transplants were comparable to those achieved with MSD in terms of both OS and EFS [61]. While graft failure is a concern with haploidentical donors, data from retrospective studies show that augmented conditioning regimens, such as 400 cGy TBI, and bone marrow grafts with higher CD34+ cell doses, improve both OS and EFS outcomes [58, 62–64]. These findings would suggest that haploidentical transplants, when supported by enhanced conditioning regimens, represent a viable and potentially superior treatment option for pediatric patients with SAA without an MSD.

This represents an alternative approach when there is a limited access to unrelated donor registries, or, when available, these donors may not be accessible in a timely manner.

## PNH: from the bench to the bedside

Therapeutic approaches in PNH and its evolution cannot be separated from what has been learned from inherited complement deficiencies. New therapeutic strategies in the era of novel complement inhibitors (CIs) are increasingly available. The complement system’s pivotal role in innate immunity is well known, particularly its involvement in pathogen elimination and cancer cell destruction [65, 66]. Inherited deficiencies in complement control proteins, such as factor H and factor I, may lead to uncontrolled activation of the alternative pathway, resulting in diseases like atypical hemolytic uremic syndrome and kidney damage [67–69]. Small molecules targeting factor B or factor D may have potential as therapeutic strategies [69–74] for conditions, such as IgA nephropathy and C3 glomerulopathy [75–78].

While complement inhibition therapies showed promising results in different clinical settings, they must be paired with immunization against bacterial infections to mitigate risks [71]. An example is the use of anti-C1s monoclonal antibodies in treating cold agglutinin disease [79]. Unlike in inherited complement deficiencies, therapies targeting C1s have proven effective in adults without causing significant complications [80, 81], highlighting the concept that therapeutic inhibition of given complement proteins is not necessarily the phenocopy of their inherited deficiency.

Over the last two decades, eculizumab, a C5 inhibitor, has had a significant impact in reducing intravascular hemolysis and improving clinical outcomes for PNH patients [82]. Despite its success, eculizumab can only address terminal complement inhibition, leaving proximal complement activation unchecked. This partial inhibition may cause extravascular hemolysis due to the opsonization of red blood cells by C3 fragments [82–85]. In response to these issues, there has been a drive to develop proximal CIs, such as the C3 inhibitor, pegcetacoplan, or factor B and factor D inhibitors, iptacopan and danicopan, respectively. These CIs target earlier phases of the complement cascade, limiting extravascular hemolysis.

Clinical trials with pegcetacoplan have demonstrated that inhibition of both intravascular and extravascular hemolysis results in improved hematological response [86–88]. Similarly, iptacopan monotherapy has shown excellent results [89], leading to improved hemoglobin concentrations. Almost superimposable results were observed with danicopan, which, in contrast, was exploited as an add-on therapy on top of a C5 inhibitor [90].

While proximal CIs demonstrated efficacy both as monotherapies and as add-on treatment to C5 inhibitors [91, 92], there are concerns about the risk of breakthrough hemolysis (BTH), particularly when treatment was interrupted.

Regardless of the therapeutic strategy employed (whether monotherapy or combination therapy), the treatment of PNH intrinsically involves a paradox: increased complement inhibition could lead to larger populations of PNH red blood cells, which in turn may heighten the BTH [87, 88, 93–98].

In addition to the obvious BTH seen in the presence of sub-therapeutic drug levels (the so-called pharmacokinetic BTH), a kind of BTH may still be observed when a drug is within its therapeutic range, rather than defined as pharmacodynamic BTH. Residual hemolysis in C5 inhibitors-treated PNH patients would be driven by the strength of complement activation and C3 deposition. Combining anti-C5 agents with proximal CIs or using proximal CIs as monotherapy may help mitigate this residual hemolysis [90, 99, 100].

Moreover, CI discontinuation is associated with increased risks of severe hemolysis: switching to another inhibitor could prevent exacerbation of the condition. As a result of increased hemolytic risk, data on monotherapy *versus* combination therapy for CIs are limited, particularly regarding pharmacokinetic/pharmacodynamic profiles and biomarkers.

The combination therapy may not necessarily improve efficacy but could enhance safety. This is especially useful in preventing BTH in patients with large PNH erythrocyte mass, in whom even transient residual complement activity may result in massive hemolysis with possibly severe clinical consequences [98].

## Conclusions

The European School of Hematology (ESH), European Bone Marrow Transplantation (EBMT), European Hematology Association (EHA), and the International PNH Interest Group (IPIG) 3rd Translational Research Conference provided invaluable insights into the latest developments in the understanding, diagnosis, and treatment of BMFSs and leukemia predisposition syndromes. The conference highlighted significant advances in stem cell biology, including the mechanisms of genotoxic stress, inflammation, and stem cell failure. Noteworthy progress was discussed in the fields of FA, ribosomal diseases, and TBD. Additionally, the exploration of novel therapeutics for idiopathic conditions, notably complement cascade modulation in PNH, and improvements in transplant management or IST in the setting of AA, showcased promising directions for future clinical application.

The advances presented at the meeting fostered top-level discussion in the field, aiming to fill the obvious gaps between basic science and direct implications for clinical hematologists. This paves the way for future research, leading to a better understanding of the biology of these diseases. Ultimately, these advances hold promise for clinical translation in order to improve the daily care of all the patients affected by BMFSs and similar conditions.
